# Wnt/β-catenin activation cooperates with loss of p53 to cause adrenocortical carcinoma in mice

**DOI:** 10.1038/s41388-020-1358-5

**Published:** 2020-06-19

**Authors:** Kleiton Silva Borges, Emanuele Pignatti, Sining Leng, Dulanjalee Kariyawasam, Gerard Ruiz-Babot, Fernando Silva Ramalho, Makoto Mark Taketo, Diana L. Carlone, David T. Breault

**Affiliations:** 1 Division of Endocrinology, Boston Children’s Hospital, Boston, MA 02115 USA; 2 Department of Pediatrics, Harvard Medical School, Boston, MA 02115 USA; 3 Department of Pediatrics, Ribeirao Preto Medical School, University of Sao Paulo, Ribeirao Preto, Brazil; 4 Division of Medical Sciences, Harvard Medical School, Boston, MA 02115 USA; 5 Department of Pathology, Ribeirao Preto Medical School, University of Sao Paulo, Ribeirao Preto, Brazil; 6 Division of Experimental Therapeutics, Graduate School of Medicine, Kyoto University, Kyoto, 606-8506 Japan; 7 Harvard Stem Cell Institute, Cambridge, MA 02138 USA

**Keywords:** Adrenal tumours, Adrenal tumours, Adrenal tumours, Adrenal tumours, Adrenal tumours

## Abstract

Adrenocortical carcinoma (ACC) is a rare and aggressive malignancy with limited therapeutic options. The lack of mouse models that recapitulate the genetics of ACC has hampered progress in the field. We analyzed The Cancer Genome Atlas (TCGA) dataset for ACC and found that patients harboring alterations in both p53/Rb and Wnt/β-catenin signaling pathways show a worse prognosis compared with patients that harbored alterations in only one. To model this, we utilized the *Cyp11b2(AS)*^*Cre*^ mouse line to generate mice with adrenocortical-specific Wnt/β-catenin activation, *Trp53* deletion, or the combination of both. Mice with targeted Wnt/β-catenin activation or *Trp53* deletion showed no changes associated with tumor formation. In contrast, alterations in both pathways led to ACC with pulmonary metastases. Similar to ACCs in humans, these tumors produced increased levels of corticosterone and aldosterone and showed a high proliferation index. Gene expression analysis revealed that mouse tumors exhibited downregulation of *Star* and *Cyp11b1* and upregulation of *Ezh2*, similar to ACC patients with a poor prognosis. Altogether, these data show that altering both Wnt/β-catenin and p53/Rb signaling is sufficient to drive ACC in mouse. This autochthonous model of ACC represents a new tool to investigate the biology of ACC and to identify new treatment strategies.

## Introduction

Adrenocortical carcinomas (ACCs) are rare tumors with a highly aggressive clinical phenotype and an annual incidence of 0.5–2 people per million worldwide [1, 2]. Approximately 35% of patients with ACC survive 5 years after diagnosis [1]. In addition, 50–75% of patients diagnosed with ACC present with excess production of steroid hormones, such as glucocorticoids and/or androgens [1, 2]. Currently, surgical resection of the primary tumor is the only curative therapy for ACC [3]. However, half of all patients with ACC are diagnosed with disseminated metastases at advanced disease stages, and around one-third of patients with localized disease at diagnosis develop postoperative metastases [1, 2].

Standard treatment for advanced ACC consists of radiotherapy and chemotherapy, including the adrenolytic agent mitotane, which have had limited impact on overall patient survival [2, 4]. The disappointing results of traditional therapies on ACC outcomes has led to the pursuit of new therapeutic approaches, which, to date, have not resulted in significant breakthroughs [5, 6]. These results are likely due, in part, to the lack of mouse models that recapitulate the genetics of ACC. Thus, there is an urgent need to develop relevant mouse models of malignant ACC in order to test new therapeutic approaches [7, 8].

The discovery and successful translation of new therapies for ACC patients will require a more comprehensive understanding of the underlying genomic, transcriptional, and epigenetic programs driving adrenocortical carcinogenesis. Such knowledge will allow for the development of in vitro and in vivo model systems that more closely resemble ACC tumor biology. Recently, genomic analyses of ACC have highlighted several genes, which when mutated appear to function as drivers of sporadic adrenocortical tumorigenesis [5, 9–11]. Gain-of-function (GOF) mutations in *CTNNB1* (the gene encoding β-catenin) and biallelic deletion or loss of function mutation in *ZNRF3* (a negative regulator of the Wnt/β-catenin pathway), which lead to constitutive activation of Wnt/β-catenin signaling, are among the most common somatic alterations in ACC [9, 10].

Despite the high frequency of mutations in Wnt/β-catenin pathway genes in human ACC, such mutations alone are insufficient to induce malignant transformation in mouse models, although they have been linked to the development of benign adrenocortical tumors [12–14]. Based on this, it is likely that additional genetic alterations are required for progression of this disease. To explore this possibility, different groups combined Wnt/β-catenin activation and overexpression of *IGF2*, a mitogen commonly altered in ACC [13, 15]. However, these mice failed to induce ACC indicating that other factors or signaling pathways, in concert with Wnt/β-catenin, drive malignant transformation within the adrenal cortex.

The p53 protein is a critical tumor suppressor and a potent transcription factor that plays a key role in maintaining genomic stability [16, 17]. Mutations in *TP53*, the gene that encodes human p53, have also been associated with ACC in both familial and sporadic forms [18, 19]. In the familial form, ACC are frequently diagnosed in Li Fraumeni Syndrome (LFS) carriers, a cancer predisposition syndrome caused by *TP53* germline mutations [19, 20]. Consistent with this, a 10-fold increase in the incidence of adrenocortical tumors has been observed in Southeastern Brazil, where a common germline mutation exists within the p53 oligomerization domain [p.R337H] [21]. Despite this, neither mice carrying a homolog of the human TP53 R337H mutation nor a global deletion of *Trp53*, the gene encoding murine p53, develop ACC [22, 23], arguing that p53 disruption alone is insufficient to induce malignant transformation in the adrenal cortex.

Recent genomic clustering analyses revealed that p53/Rb and Wnt/β-catenin are the leading pathways altered in the ACC molecular subgroup with the poorest outcome [9–11], suggesting that these pathways together play a significant role in the tumorigenesis of ACC. Mutations in the *TP53* and *CTNNB1* genes are among the most recurrent genetic alterations associated with disruption of these pathways in the ACCs [9, 10]. Here, we demonstrate that the combination of adrenal-specific β-catenin GOF and p53 deletion in mice resulted in metastatic ACC that produce corticosteroids in excess, a key feature of human ACC. This mouse strain extends our understanding of the spectrum of molecular causes of this disease and provides an autochthonous model to investigate ACC biology and to test new therapeutic targets.

## Results and discussion

### Human ACCs containing genetic alterations in both Wnt/β-catenin and p53/Rb pathways show a worse prognosis

**Fig. 1:**
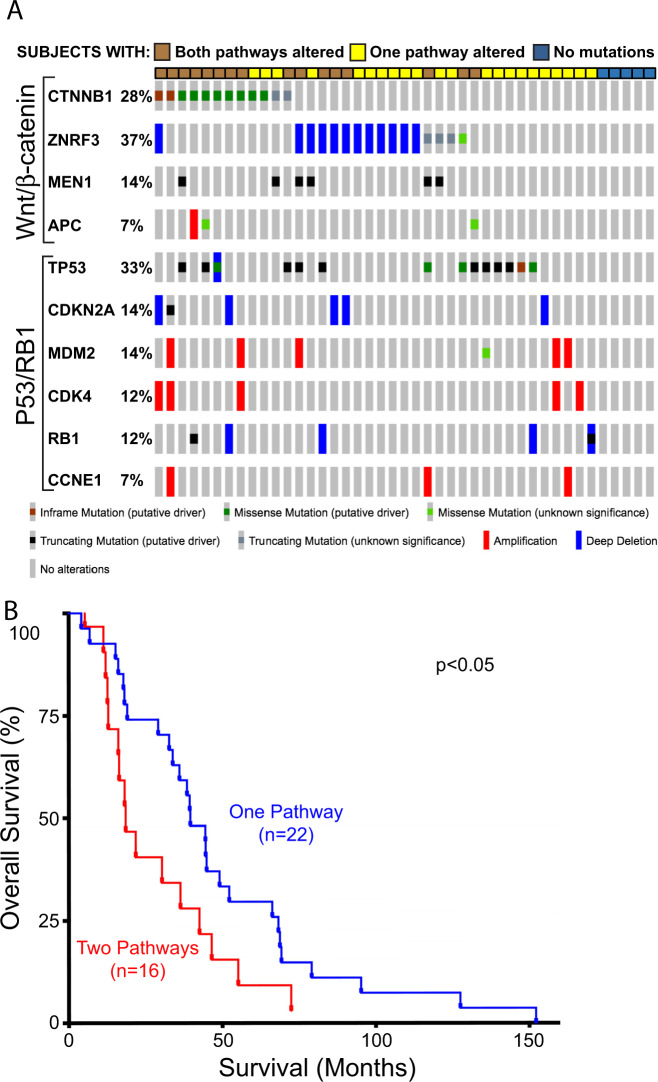
Alterations in human ACC of Wnt/β-catenin and P53/RB1 network genes in the C1A molecular subgroup. **a** Analysis of 43 ACC samples from cBioPortal showing mutations and copy number gains and losses of indicated genes. Somatic alterations of *CTNNB1*, *ZNFR3*, *MEN1*, and *APC* genes result in modification of the Wnt/β-catenin pathway and somatic alterations in *TP53*, *CDKN2A*, *MDM2*, *CDK4*, *RB1*, and *CCNE1* genes affect the p53 apoptosis/Rb1 cell cycle pathway [10]. 88% (38/43) of human ACCs from C1A subgroup harbor at least one altered gene from either pathway, with 37% (16/43) harboring altered genes in both pathways. 12% (5/43) of the patients do not harbor somatic mutations in any of these genes. **b** Kaplan–Meier analysis showing overall disease-free survival for the set of patients with alterations in one pathway (blue) versus those that show alterations in both pathways (red). *P* < 0.0001. Log-rank (Mantel–Cox) test.

To gain further insight into the genetic changes that arise in human ACC, we reanalyzed genomic data from the ACC TCGA dataset using cBioPortal [10, 24]. Previously, unsupervised clustering analysis identified two unique transcriptional subtypes of ACC, C1A (CoCII-III) and C1B (CoCI) [9, 10]. The C1A subtype is comprised of more aggressive tumors and shows enrichment for tumors with somatic mutations in genes linked to the Wnt/β-catenin and p53/Rb pathways [9, 10]. Thus, we focused our analysis on patients from this group (*n* = 43). Analysis of these tumors identified alterations in the following genes linked to these pathways: *CTNNB1*, *ZNFR3*, *MEN1*, and *APC* (Wnt/β-catenin pathway) and *TP53*, *CDKN2A*, *MDM2*, *CDK4*, *RB1*, and *CCNE1* (p53/Rb pathway). Specific mutations are described in Table S1. Most ACCs from the C1A subgroup (88%) harbor at least one altered gene from either pathway, with 37% harboring altered genes in both pathways (Fig. 1a). Confirming these results, similar frequencies were observed in a reanalysis of the ENSAT genomic data [9], an independent cohort of patients with ACCs (Table S2). Of interest, patients with ACCs harboring alterations in genes from both pathways showed an overall lower survival rate compared with patients harboring alterations in only one pathway (Fig. 1b), arguing that the combination of mutations in these two pathways could play a critical role in the development of aggressive ACCs.

### Alterations in p53 and Wnt signaling lead to ACA and ACC in mice

To investigate the effect of Wnt/β-catenin activation and p53/Rb dysfunction in the adrenal cortex, we crossed the adrenal-specific Aldosterone Synthase (*AS*) *AS*^*Cre/+*^ mouse strain [25], in which Cre expression is first noted within the zona Glomerulosa (zG) around the time of birth, with mice harboring the conditional p53 Loss-of-function (p53-LOF) allele *Trp53*^*flox/flox*^ [26] and/or the conditional β-catenin GOF (βcat*-*GOF) allele *Ctnnb*^*flox(ex3)/+*^ [27]. These crosses led to the generation of the following adrenal-specific transgenic mice: (1) p53*-*LOF (*AS*^*Cre/+*^:: *Trp53*^*flox/flox*^), PCre^AS/+^; (2) βcat-GOF (*AS*^*Cre/+*^:: *Ctnnb*^*flox(ex3)/+*^), BCre^AS/+^; and (3) p53-LOF/βcat-GOF (*AS*^*Cre/+*^:: *Trp53*^*flox/flox*^:: *Ctnnb*^*flox(ex3)/+*^), BPCre^AS/+^. *AS*^*Cre/+*^ mice served as controls.

**Fig. 2:**
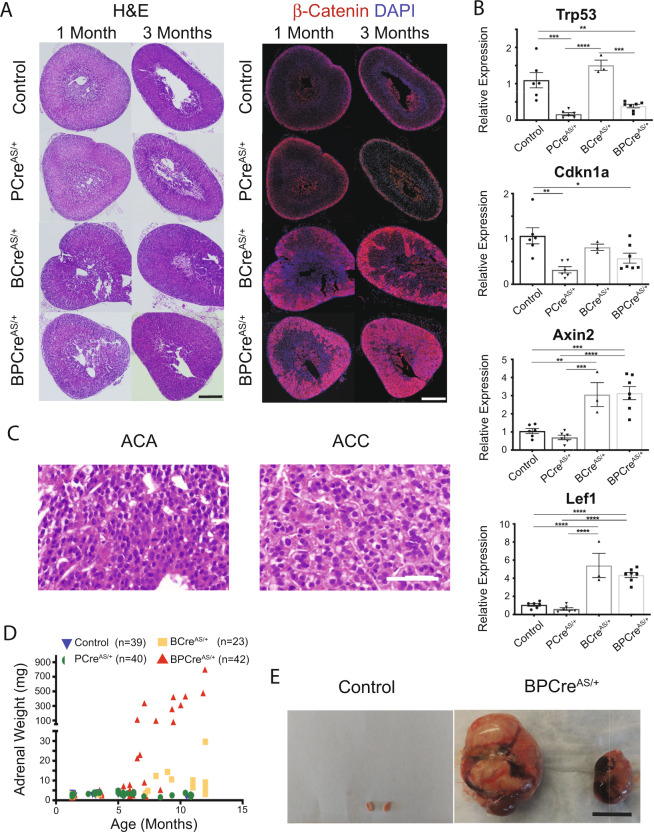
Combined activation of Wnt/β-catenin and deregulation of p53/RB1 pathway causes adrenocortical neoplastic transformation in mice. **a** Histological analysis of the adrenal phenotype. Hematoxylin/eosin (H&E) and β-catenin staining of Controls, PCre^AS/+^, BCre^AS/+^, and BPCre^AS/+^ adrenals at 1 month (*n* = 4, *n* = 4, *n* = 4, *n* = 5, respectively) and 3 months (*n* = 6, *n* = 5, *n* = 3, *n* = 7, respectively). All data shown are from female mice. Scale bar: 500 μm. **b** Quantitative representation of mRNA expression of genes encoding *Trp53*, *Cdkn1a*, *Axin2*, and *Lef1* in Control (wild-type), PCre^AS/+^, BCre^AS/+^, and BPCre^AS/+^ adrenals at 3 months. Bars represent the mean ± SEM. *P < 0.05, **P < 0.01, ***P < 0.001, by one-way ANOVA followed by the Bonferroni test. All data shown are from female mice. **c** Images of H&E staining of a benign adrenocortical adenoma (ACA) (Weiss = 1) and a malignant adrenocortical carcinoma (ACC) (Weiss = 3) from female mice at 3 months of age. Scale bars, 50 μm. **d** Evaluation of adrenal weight over time from Control, PCre^AS/+^, BCre^AS/+^, and BPCre^AS/+^ mice. BPCre^AS/+^ mice show an increased tissue weight over one year compared to controls, PCre^AS/+^, and BCre^AS/+^ mice. Data shown are from male and female mice. **e** Gross adrenal anatomy in 10-month-old control (left) and BPCre^AS/+^ (right) female mice. Scale bars, 1000 μm.

Histological analysis was employed to grossly assess tissue morphology using hematoxylin and eosin (H&E) staining. In addition, we used immunofluorescent (IF) staining for β-catenin (a zG-specific marker) [28] to assess changes in the zG (Fig. 2a, S1a). Analysis of adrenals from PCre^AS/+^ mice revealed no gross morphological differences at 1 (*n* = 7) or 3 (*n* = 6) months of age, compared with controls, with a normal appearing zG and zona fasciculata (zF). Consistent with this, β-catenin IF revealed no difference in zG morphology and was comparable with controls (Fig. 2a). Analysis of gene expression in whole adrenals from PCre^AS/+^ mice at 3 months of age, compared with controls, showed low levels of *Trp53* expression and its main downstream gene target *Cdkn1a* [29] (Fig. 2b), confirming deletion of *Trp53*.

Histological and morphological analyses of BCre^AS/+^ mice demonstrated progressive expansion of the zG at 1 (*n* = 8) and 3 (*n* = 6) months of age, with a corresponding expansion of the β-catenin^+^ domain (Fig. 2a, S1a), consistent with our recent report [30]. Furthermore, these changes were accompanied by an increase in the expression of Wnt/β-catenin target genes *Axin2* and *Lef1* [13] (Fig. 2b).

Histological and IF analysis of adrenals from BPCre^AS/+^ mice at 1 (*n* = 5) and 3 (*n* = 7) months of age revealed an even greater expansion of the β-catenin^+^ domain compared with BCre^AS/+^, PCre^AS/+^, and controls, suggesting an enhanced hyperplastic response (Figs. 2a and S1a). Moreover, as expected, gene expression analysis of BPCre^AS/+^ adrenals revealed increased levels of *Axin2* and *Lef1* and decreased levels of *Trp53* and *Cdkn1a*, compared with controls (Fig. 2b).

Interestingly, we observed nodular masses in ~30% of BPCre^AS/+^ adrenals (2 of 7) at 3 months of age (Figs. 2c and S1b). We then applied the Weiss histological classification criteria to establish whether these nodules were benign adrenocortical adenomas (ACA, Weiss Score < 3) or malignant ACC (Weiss ⩾ 3) [31]. One nodule demonstrated pleomorphic and hyperchromic nuclei and frequent mitotic figures along with some atypical mitoses, consistent with malignant ACC (Figs. 2c and S1b). In contrast, the second nodule showed low nuclear pleomorphism and discrete mitotic figures, with no atypical mitoses or necrotic foci, consistent with a benign ACA (Figs. 2c and S1b). Taken together, these results indicate that the combination of β-catenin GOF and p53 LOF is sufficient to induce adrenal tumor formation by 3 months.

To gain further insight into tumor progression in this model, we next analyzed adrenal weight from control, PCre^AS/+^, BCre^AS/+^, and BPCre^AS/+^ mice from 5 weeks to 12 months of age. Non-linear regression analysis of average adrenal weights (per animal) over time revealed an exponential rate of adrenal growth for BPCre^AS/+^ mice (~200-fold increase at 12 months) compared with control mice (Fig. 2d, e). On closer analysis, no differences were noted between control and PCre^AS/+^ mice, whereas a modest ~3-fold increase was observed in BCre^AS/+^ mice at 6–12 months of age (Table S3).

**Fig. 3:**
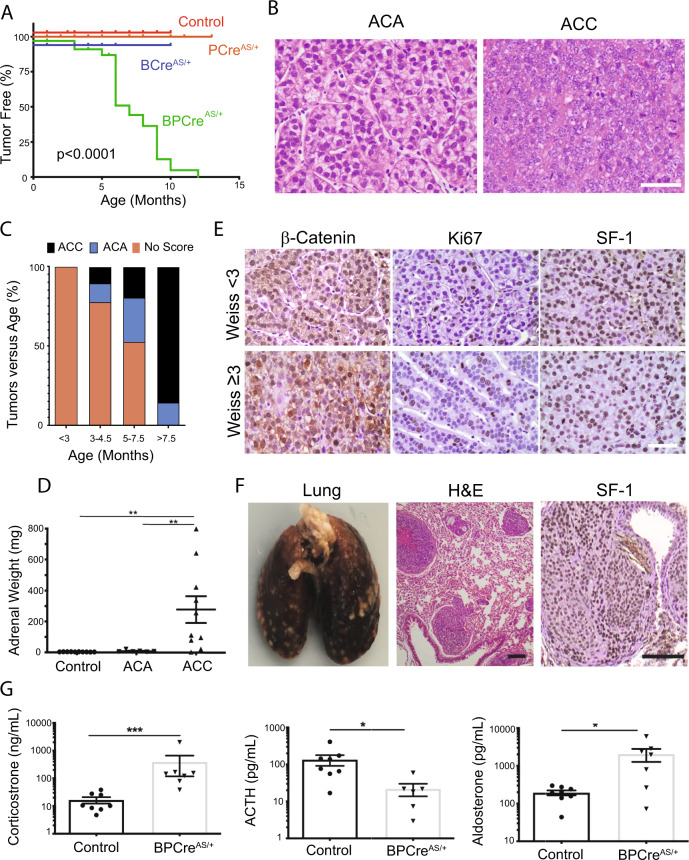
Wnt/β-catenin activation combined with *Trp53* deletion induces the evolution of adrenocortical malignant disease in mice. **a** Kaplan–Meier analysis showing the percent tumor free in Controls, PCre^AS/+^, BCre^AS/+^, and BPCre^AS/+^ mice. Only BPCre^AS/+^ mice give rise to adrenocortical tumors, *P* < 0.0001, Log-rank (Mantel–Cox). Data shown are from male and female mice. **b** Examples of Hematoxylin/eosin (H&E) staining of ACA (Weiss = 2, 6.5 month) and ACC (Weiss = 6, 10 months) tumors from female mice. Scale bar = 50 µm. **c** Histologic progression of adrenocortical tumors using the Weiss criteria. Percentages of BPCre mice with adenomas (ACA), carcinomas (ACC) or no score at age <3 months (*n* = 11), 3-4.5 months (n = 9), 5-7.5 months (*n* = 15), and >7.5 months (*n* = 7). Data from male and female mice. **d** Average adrenal weight from Control (*n* = 10) versus ACA (*n* = 6) or ACC (*n* = 10) from BPCre^AS/+^ mice from 3 to 12 months of age. Mean weight ± SEM **P < 0.01, Mann–Whitney test. **e** IHC staining against the indicated proteins in ACA and ACC tumors. Scale bar = 50 μm. **f** Macroscopic (left) and microscopic (center, right) images of lung from a BPCre^AS/+^ mouse. H&E and SF-1 IHC show discrete metastatic nodules. Scale bar = 100 μm. **g** Quantitative analysis of plasma corticosterone, ACTH, and aldosterone from Control mice (*n* = 6) and BPCre^AS/+^ mice with ACC. Bars represent the mean ± SEM from Control (*n* = 8) and BPCre^AS/+^ (*n* = 6–7). Data shown are from male and female mice at 8–9 months of age. Student’s *t*-test, *P < 0.05, **P < 0.01, ***P < 0.001.

Next, we employed the Weiss classification criteria to determine whether the dramatic increase in adrenal weight correlates with the presence of benign ACA or malignant ACC. Adrenals from 1 to 12 months old mice showed no evidence for ACA or ACC for control (*n* = 45), PCre^AS/+^ (*n* = 41), and BCre^AS/+^ (*n* = 39) mice (Fig. 3a). In contrast, analysis of BPCre^AS/+^ (*n* = 42) mice revealed the presence of adrenocortical tumors (ACA and ACC) in 38% (16/42) of all mice analyzed (Figs. 3a, b and S1c) (Tables S4 and S5). Further analysis revealed increasing tumor frequency with age, with 0% (0/11) of mice <3 months of age, 22% (2/9) of mice between 3 and 4.5 months of age, 47% (7 of 15) of mice 5–7.5 months of age, and 100% (7 of 7) of mice >7.5 months of age developing tumors (Fig. 3c) (Table S6). Moreover, in mice 3–7.5 months old, ~45% of tumors analyzed were ACC, whereas in the older group (>7.5 months) 86% of tumors were ACC (Fig. 3c) (Table S6). Altogether, these findings show that targeted activation of the Wnt/β-catenin pathway combined with deletion of p53 in the adrenal cortex leads to ACA and ACC with 100% penetrance by 12 months of age. Finally, although human ACC is twice as likely to occur in females than in males [1] we did not observe any clear sex difference in the timing of tumor onset (Fig. S2a).

Additional analysis of the tumors showed consistent similarities between the BPCre^AS/+^ mouse model and the human adrenocortical tumors. Adrenals harboring ACC (*n* = 10) were significantly heavier compared to those with ACA (*n* = 6) and to age-matched control adrenals (*n* = 6) (Fig. 3d). Also, immunohistochemical (IHC) analysis of ACC (*n* = 9) and ACA (*n* = 5) showed clear evidence of nuclear and cytoplasmic accumulation of β-catenin [32] and a high Ki67-labeling index (ranging from 7% to 45%) [33] (Fig. 3e) (Table S5). Moreover, IHC staining for SF-1 confirmed the steroidogenic origin of these tumors (Fig. 3e) [34]. Together, these data demonstrate that the mouse tumors recapitulate important pathological aspects of the human disease.

### Combined p53-LOF and βcat-GOF lead to metastatic and hormonally active ACC

Approximately 25–30% of patients with ACC present with metastatic disease and most patients with ACC present with excess production of adrenocortical hormones [1, 2]. Thus, to assess the frequency of metastases in BPCre^AS/+^ mice we scored for macroscopic disease burden at the time of necropsy. As expected, in mice with histological evidence for ACA, no metastatic masses were detected. In contrast, 40% (4 of 10) of 7–12-month-old mice with ACC showed metastases to the lungs (Fig. 3f) (Table S5). Additionally, we analyzed plasma levels of corticosterone, ACTH, and aldosterone from mice with ACC and control mice. Mice with ACC showed an increase in both aldosterone and corticosterone levels with a corresponding decrease in ACTH levels (Fig. 3g). Consistent with recent reports [30, 35], BCre^AS/+^ mice exhibited mildly elevated levels of plasma aldosterone in older mice. No difference in corticosterone levels was observed in BCre^AS/+^ or PCre^AS/+^ mice compared with controls (Fig. S2d). Hypercortisolism is the most common endocrinopathy identified in ACC patients, affecting ~60% of patients with ACC and is associated with a poor prognosis [1, 10, 36]. By comparison, aldosterone secretion is less frequently seen in patients with ACC affecting up to ~15% of patients [1, 36, 37]. Taken together, these data indicate that ACC arising in BPCre^AS/+^ mice share these key features with human ACC.

### ACC in mice exhibit similar gene expression profiles to ACC in humans

**Fig. 4:**
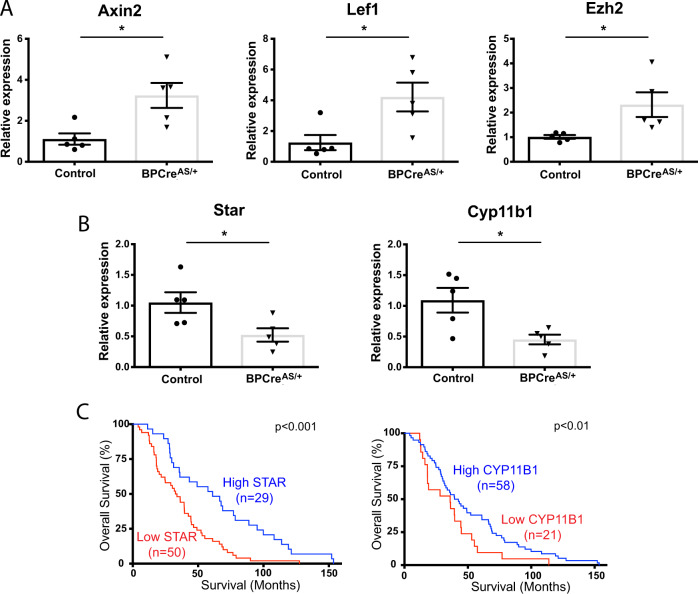
ACC in mice exhibit similar gene expression profiles to ACC in humans. **a** Analysis reveals increased gene expression for *Axin2, Lef1*, and *Ezh2* in BPCre^AS/+^ mice (*n* = 5) with ACC compared to Control mice (*n* = 5). **b** Analysis reveals decreased gene expression for *Star* and *Cyp11b1* in BPCre^AS/+^ mice (*n* = 5) compared to Control mice (*n* = 5). **c** *STAR* and *CYP11B1* expression predicts a worse prognosis in human ACC. All gene expression data are from female mice at 8–9 months of age. Statistical analyses were conducted by Student’s *t*-test **a** and **b** or by Log-rank (Mantel–Cox) **c**. *P < 0.05, **P < 0.01, ***P < 0.001.

Having established that multiple pathological and histological similarities exist between mouse and human ACC, we next compared the expression of key genes between ACC in mice and humans. Human ACC harboring alterations in the Wnt/β-Catenin pathway have previously shown increased expression of *AXIN2* and *LEF1* [9, 10] and β-catenin status in ACC correlates with a gene signature rich in TCF/LEF target genes [13]. Consistent with this, ACC arising in aged mice (8–9 months of age) also demonstrated increased expression of *Axin2* and *Lef1* (Fig. 4a). As expected, we also observed a significant decrease in *Trp53* expression in ACC arising in these aged mice (Fig. S2c). Next, we assessed the expression of *EZH2*, a histone methyltransferase which is overexpressed in human ACC and associated with disease progression, poor prognosis and inactivating mutations in the p53/Rb pathway [38]. Analysis of ACC arising in aged mice showed an increase in *Ezh2* expression (Fig. 4a, S2c), recapitulating another key finding from patients in the C1A subgroup of ACC.

*IGF2* is overexpressed in up to 90% of all human ACCs; however, studies designed to inhibit its receptor have failed to demonstrate efficacy in patients with ACC [39, 40]. Moreover, overexpression of *Igf2* in mice is not associated with progression to ACC [13, 15]. In fact, analysis of *Igf2* expression levels in ACC from BPCre^AS/+^ aged mice showed reduced expression compared with control mice (Fig. S2b). Although it has been long known that *IGF2* gene expression levels are altered in human ACCs, based on our data and previous findings, more studies are necessary to understand the role of Igf2 in ACC tumorigenesis in mice. Finally, we examined the expression of two key genes involved in steroidogenesis *STAR* and *CYP11B1*, which have been shown to be reduced in human ACC [41, 42]. Consistent with this, in ACC from aged mice we found both *Star* and *Cyp11b1* expression to be reduced compared with control adrenals (Figs. 4b and S2c). To further assess the clinical relevance of reduced expression of these genes, we analyzed TCGA dataset of ACC samples (*n* = 79). In agreement with previous results [41, 42], lower expression of *STAR* and *CYP11B1* was associated with lower overall survival of ACC patients (Fig. 4c). Taken together, these data substantiate an inverse correlation between *STAR* and *CYP11B1* expression levels with progression of ACC and raise the possibility that the BPCre^AS/+^ model could be used to study the role of these genes in ACC tumor biology. Interestingly, with the exception of Wnt signaling, all gene expression changes were specific for BPCre^AS/+^ mice with ACC when compared with PCre^AS/+^ and BCre^AS/+^ mice (Fig. S2c).

## Concluding remarks

This study describes an autochthonous mouse model of ACC that accurately recapitulates key cellular and molecular features of human ACC. BPCre^AS/+^ mice gradually develop adrenocortical tumors with increasing aggressiveness and demonstrate pathological characteristics described for human ACC, including metastasis and excess hormone production. Here, we also show that loss of the p53/Rb pathway in concert with Wnt/β-catenin activation is sufficient to initiate tumor formation, suggesting that disrupting the tumor suppressor function of the p53/Rb pathway contributes greatly to Wnt/β-catenin-induced oncogenesis in the adrenal cortex.

The p53/Rb pathway is composed of proteins that control cell cycle entry at G1 and dysregulation of these molecules is thought to lead to uncontrolled cell proliferation in ACC [43, 44]. On the other hand, alterations in proteins of the Wnt/β-catenin-signaling pathway cause aberrant Wnt activation and affect adrenocortical cell renewal, differentiation, and proliferation [12, 14, 30]. Interestingly, the combination of mutations affecting both *TP53* and *CTNNB1* have been described in a small group of both pediatric and adult patients presenting with malignant adrenocortical tumors and correlates with poor prognosis [6, 9, 10, 45, 46], though, in general, the majority of high-risk patients do not simultaneously harbor mutations in both genes [47]. Taken together, we believe that the BPCre^AS/+^ mouse model serves as an important tool to investigate the pathobiology underlying how the p53/Rb and Wnt/β-catenin pathways interact to induce adrenocortical tumorigenesis.

A recent study showed that adrenocortical specific expression of SV40 large T-antigen (AdTAg mice) (driven by a 0.5 kb region of the *Akr1b7* promoter) leads to ACC in mice [48]. In addition to inhibiting p53/Rb, it is also important to bear in mind that SV40 large T antigen impacts a number of host proteins, including Hsc70, CBP/p300, Cul7, IRS1, Fbxw7, and Bub1, which could impact tumorigenesis in this model [49]. In addition, while not directly targeted, this model also showed Wnt pathway activation, likely due to downregulation of secreted frizzled related proteins (Sfrp) and the E3 ubiquitin ligase Znrf3, which act as inhibitors of WNT signaling [48].

In summary, we have demonstrated that Wnt/β-catenin activation cooperates with loss of p53 to promote murine ACC tumorigenesis, which mimics the cardinal features of human ACC. This model will be useful for detailed analysis of tumorigenic mechanisms and clinical manifestations of ACC, contributing to the identification of biomarkers and new therapeutic targets as to preclinical testing of drugs for the treatment of ACC.

## Material and methods

### Genomic and survival analyses

TCGA data was analyzed using the cBioPortal for Cancer Genomics [10, 24].

### Mice

Experiments were performed with approval by the IACUC.

### Gene expression

Relative expression was assessed using the 2^−ΔΔCT^ method.

### Histology

Adrenals were analyzed as previously described [25, 30, 35].

### Hormone measurements

Radioimmunoassays were performed as described previously [25].

### Statistics

Two-tailed Student’s *t*-test or one-way ANOVA followed by Tukey’s analysis was performed using Graphpad Prism 8; data presented as mean ± SEM with significance *P* < 0.05.

## Supplementary information

Supplemental Materials

Supplemental Tables
