# Supplementary material for: Wnt/β-catenin activation cooperates with loss of p53 to cause adrenocortical carcinoma in mice
Source: Oncogene. 2020 Jun 19;39(30):5282–91. doi: 10.1038/s41388-020-1358-5 (PMC7378041; doi:10.1038/s41388-020-1358-5)
Supplement: Supplementary file 1 — Supplemental Materials [file 41388_2020_1358_MOESM1_ESM.docx]

**Material and Methods**

**Genomic and survival analyses**

Analysis of genomic data from 43 ACCs (C1A molecular group) and Kaplan-Meier survival analyses using data from The Cancer Genome Atlas (TCGA) dataset were conducted using the online tools of the cBioPortal for Cancer Genomics (http://www.cbioportal.org) [10,25]. The Cancer Genome Atlas gene expression profile of *CYP11B1* and *STAR* and their corresponding association with survival probability in 79 patients with ACC were obtained from the “R2: Genomics analysis and visualization platform” [http://r2.amc.nl].

**Mice**

All experiments were carried out in accordance with protocols approved by the Boston Children’s Hospital IACUC. Mice were maintained and bred on a mixed genetic background. Adrenals were cleaned of excess fat, weighed, and frozen in liquid nitrogen or fixed, as described. Adrenal weights were compared based on the mean of both adrenal glands from a single mouse.

**RNA extraction and quantitative real-time PCR (qRT-PCR)**

Total RNA was extracted from whole adrenals using the Trizol® reagent (Gibco BRL, Life Technologies®, Carlsbad, CA, USA). Complementary DNA (cDNA) was generated using the High Capacity® kit (Applied Biosystems®, Foster City, CA, USA) according to the manufacturer’s instructions. Expression levels were assessed using TaqMan® probes (Applied Biosystems) in the QuantStudio 12k Flex system (Applied Biosystems, Foster City, CA, USA). Probe information is listed in **Table S7**. Relative expression was calculated using the 2-ΔΔCT method with two internal ‘control’ genes normalized to control adrenals.

**Histology**

Adrenals were fixed in 4% paraformaldehyde for 1h at 4°C, paraffin-embedded, and cut into 5-µm sections. Antibody information and dilutions are listed in **Table S8**. For IF, slides were unmasked with 10mM NaCitrate, pH 6.0 + 0.05% Tween-20 and blocked for 1 h at room temperature followed by primary antibody incubation overnight at 4°C. Primary antibodies were detected with secondary antibodies (1:500; Invitrogen). Nonspecific staining was blocked using 10% normal goat serum (NGS) and 1% bovine serum albumin (BSA) in TBST and nuclei were counterstained with DAPI. Slides were mounted using ProLong Gold (Life Technologies). IHC was performed on sections of FFPE using the polymer-based methodology, the Reveal—Biotin-Free Polyvalent DAB kit (Spring, cat. N. SPD-15, CA, USA). Quantification of Ki67 was performed using ImageJ (version 1.48). The average of 10 images per adrenal were analyzed. Images were acquired on a Nikon TS2 microscope with a Nikon camera DSFi3.

**Ki67 Labeling Index**

The Ki67 Labeling Index was determined using IHC as previously described [33]. Briefly, 5 to 10 high power fields were selected, and at least 1,000 cells were evaluated. The labeling index represents the number of Ki67 positive cells per 100 adrenocortical cells. Tissues with more than 5% Ki67 positive cells were considered hyperproliferative.

**Hormone measurements**

Plasma collection and hormone measurements by radioimmunoassay were performed as described previously [26].

**Statistics**

All sample size (n) values used for statistical analyses are provided in the relevant figures and supplementary figures. Differences in tumor onset between different mouse genotypes and sexes were assessed using the Log-rank Mantel-Cox test. For comparison of two groups, a two-tailed Student's t-test was performed. For groups of three or more, one-way ANOVA followed by Tukey's post hoc analysis was performed. Data are presented as the mean ± SEM. Statistical significance was set at p < 0.05. Statistical analyses were performed using Graphpad Prism 8.

**
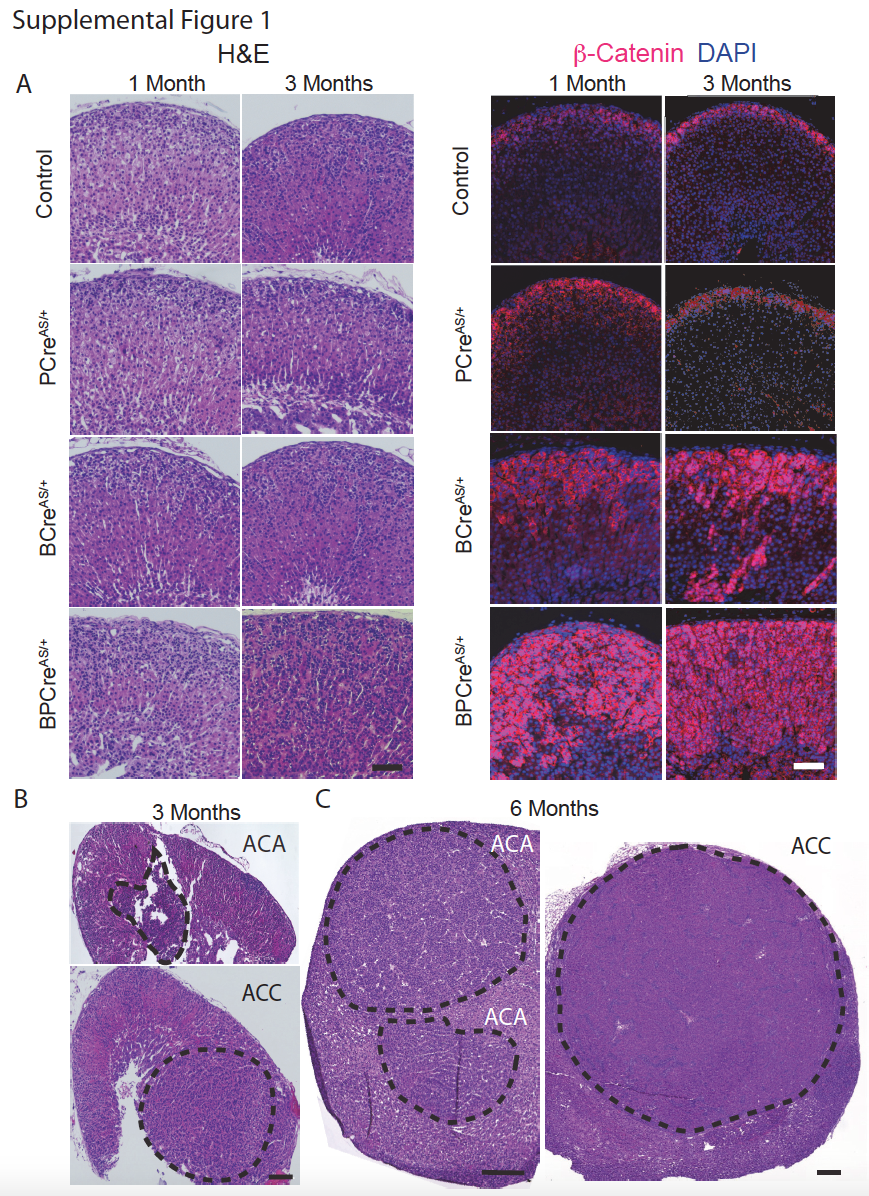
**

**Supplementary Figure 1. (A)** Histological analysis of the adrenal phenotype. High power images of hematoxylin/eosin (H&E) and β-catenin staining of Controls, PCre^AS/+^, BCre^AS/+^ and BPCre^AS/+^  adrenals at 1 month (n=4, n=4, n=4, n=5, respectively) and 3 months (n=6, n=5, n=3, n=7, respectively). Scale bar: 50 μm. All data shown are from female mice. (**B**) Lower magnification images of H&E staining of adrenocortical nodules with a benign Adrenocortical Adenoma (ACA) (Weiss = 1) and a malignant Adrenocortical Carcinoma (ACC) (Weiss = 3) from female mice at 3 months of age. Scale bars: 200 μm. (**C**) Lower magnification images of H&E staining of a benign Adrenocortical Adenoma (ACA) (Weiss = 1) (left panel) and a malignant Adrenocortical Carcinoma (ACC) (Weiss = 5) (right panel) from female mice at 6 months of age. Scale bars: 200. The dotted line indicates the tumor area.

**
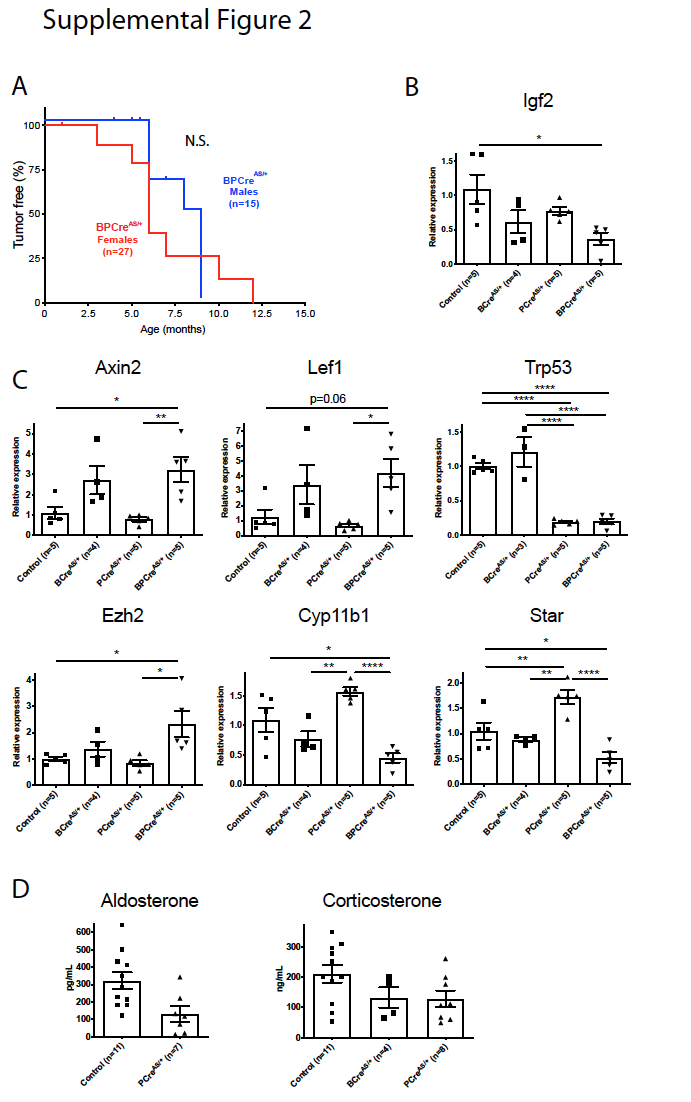
**

**Supplementary Figure 2.** **(A)** Kaplan-Meier analysis showing the percent tumor free curves in BPCre^AS/+^ male and female mice. No statistical difference was found between the groups. Log-rank (Mantel-Cox). **(B)** *Igf2* expression is downregulated in the BPCre^AS/+^ mice compared to control adrenals. Control (n=5) and BPCre^AS/+^ (n=5). Gene expression data are from female mice at 8-9 months of age. *P<0.05, Student's t-test. **(C)** Quantitative representation of the expression of genes encoding, Axin2, Lef1, Cyp11b1, Star, Ezh2, Igf2 and Trp53 in Control (wild-type) (n=5), PCre^AS/+^ (n=4), BCre^AS/+^ (n=5) adrenals and BPCre^AS/+^ tumors at 8 months (n=5). Bars represent the mean±SEM. *P < 0.05, **P < 0.01, ***P < 0.001, One-way ANOVA followed by the Bonferroni test. All data shown are from female mice. **(D)** Quantitative analysis of plasma corticosterone and/or aldosterone from 3-month-old female mice: Control (wild-type) (n=11), PCre^AS/+^ (n=4) and BCre^AS/+^ (n=7) mice. Bars represent the mean±SEM. *P < 0.05, **P < 0.01, ***P < 0.001, One-way ANOVA followed by the Tukey's post hoc.
