# Supplementary material for: Wnt/β-catenin activation cooperates with loss of p53 to cause adrenocortical carcinoma in mice
Source: Oncogene. 2020 Jun 19;39(30):5282–91. doi: 10.1038/s41388-020-1358-5 (PMC7378041; doi:10.1038/s41388-020-1358-5)
Supplement: Supplementary file 2 — Supplemental Tables [file 41388_2020_1358_MOESM2_ESM.pdf]

Table S1 – List of gene mutations in patients from the C1A ACC subgroup. Data from Zheng et al., 2016.

Wnt/β-catenin genes	p53/Rb genes	Both pathways	One pathway	No mutations

Sample ID	Patient ID	CTNNB1: AMP HOMDEL MUT FUSION;	APC: AMP HOMDEL MUT FUSION;	MEN1: AMP HOMDEL MUT FUSION;	ZNRF3: AMP HOMDEL MUT FUSION;	TP53: AMP HOMDEL MUT FUSION;	CDK4: AMP HOMDEL MUT FUSION;	CDKN2A: AMP HOMDEL MUT FUSION;	RB1: AMP HOMDEL MUT FUSION;	MDM2: AMP HOMDEL MUT FUSION;	CCNE1: AMP HOMDEL MUT FUSION;
TCGA-OR-A5J2-01	TCGA-OR-A5J2	MUT: Y30*;				MUT: H168Cfs*8;					
TCGA-OR-A5JY-01	TCGA-OR-A5JY	MUT: S45P;					CNA: AMP;			CNA: AMP;	
TCGA-OR-A5LE-01	TCGA-OR-A5LE	MUT: S45P;	MUT: N125S;			MUT: P27Lfs*17;					
TCGA-OR-A5JJ-01	TCGA-OR-A5JJ	MUT: S45F;				MUT: C275S; CNA: HOMDEL;					
TCGA-OR-A5JM-01	TCGA-OR-A5JM	MUT: S45del;					CNA: AMP;	MUT: A30Gfs*18;		CNA: AMP;	CNA: AMP;
TCGA-OR-A5K9-01	TCGA-OR-A5K9	MUT: S45_P52del;			CNA: HOMDEL;		CNA: AMP;	CNA: HOMDEL;			
TCGA-OR-A5K5-01	TCGA-OR-A5K5	MUT: G34V;						CNA: HOMDEL;	CNA: HOMDEL;		
TCGA-OR-A5JS-01	TCGA-OR-A5JS	MUT: G34R;	CNA: AMP;						MUT: Q217*;		
TCGA-OR-A5LJ-01	TCGA-OR-A5LJ	MUT: G34E;		MUT: R521Pfs*15;		MUT: R213*;					
TCGA-OR-A5J5-01	TCGA-OR-A5J5				MUT: R307W;	MUT: R273C;					
TCGA-OR-A5JA-01	TCGA-OR-A5JA			MUT: X267_splice;	CNA: HOMDEL;	MUT: E56*,X25_splice;				CNA: AMP;	
TCGA-OR-A5JW-01	TCGA-OR-A5JW				CNA: HOMDEL;			CNA: HOMDEL;			
TCGA-OR-A5KO-01	TCGA-OR-A5KO				CNA: HOMDEL;	MUT: E339*;			CNA: HOMDEL;		
TCGA-OR-A5KY-01	TCGA-OR-A5KY			MUT: R420*;	MUT: L191Qfs*19;	MUT: C135Y;					CNA: AMP;
TCGA-OR-A5LC-01	TCGA-OR-A5LC				CNA: HOMDEL;			CNA: HOMDEL;			
TCGA-PK-A5HB-01	TCGA-PK-A5HB		MUT: R2543K;			MUT: X307_splice;					
TCGA-OR-A5L3-01	TCGA-OR-A5L3	MUT: S45P;									
TCGA-OR-A5L8-01	TCGA-OR-A5L8	MUT: S45P;									
TCGA-OR-A5K6-01	TCGA-OR-A5K6	MUT: A39Efs*3;		MUT: E364Vfs*6;							

TCGA-OR-A5J1-01	TCGA-OR-A5J1									CNA: AMP;	CNA: AMP;
TCGA-OR-A5J8-01	TCGA-OR-A5J8					MUT: T125=;					
TCGA-OR-A5J9-01	TCGA-OR-A5J9				CNA: HOMDEL;						
TCGA-OR-A5JB-01	TCGA-OR-A5JB					MUT: Y234C;			CNA: HOMDEL;		
TCGA-OR-A5JE-01	TCGA-OR-A5JE								MUT: E458*; CNA: HOMDEL;		
TCGA-OR-A5JF-01	TCGA-OR-A5JF				CNA: HOMDEL;						
TCGA-OR-A5JG-01	TCGA-OR-A5JG					MUT: X125_splice;				MUT: E430Q;	
TCGA-OR-A5JP-01	TCGA-OR-A5JP					MUT: E339Afs*8;					
TCGA-OR-A5K2-01	TCGA-OR-A5K2				CNA: HOMDEL;						
TCGA-OR-A5KT-01	TCGA-OR-A5KT						CNA: AMP;			CNA: AMP;	
TCGA-OR-A5KW-01	TCGA-OR-A5KW			MUT: Y317*;	CNA: HOMDEL;						
TCGA-OR-A5KZ-01	TCGA-OR-A5KZ					MUT: E180_D184del;					
TCGA-OR-A5LB-01	TCGA-OR-A5LB				MUT: V394Sfs*4;						
TCGA-OR-A5LD-01	TCGA-OR-A5LD						CNA: AMP;				
TCGA-OR-A5LG-01	TCGA-OR-A5LG							CNA: HOMDEL;			
TCGA-OR-A5LO-01	TCGA-OR-A5LO				CNA: HOMDEL;						
TCGA-OR-A5LS-01	TCGA-OR-A5LS				CNA: HOMDEL;						
TCGA-OU-A5PI-01	TCGA-OU-A5PI				CNA: HOMDEL;						
TCGA-P6-A5OF-01	TCGA-P6-A5OF			MUT: W270*;	MUT: E339*,X339_splice;						
TCGA-OR-A5J3-01	TCGA-OR-A5J3										
TCGA-OR-A5J7-01	TCGA-OR-A5J7										
TCGA-OR-A5K0-01	TCGA-OR-A5K0										
TCGA-OR-A5KV-01	TCGA-OR-A5KV										
TCGA-OR-A5KX-01	TCGA-OR-A5KX										

Table S2 – Frequency of patients with genetic alterations in Wnt/β-catenin and p53/Rb pathways in the C1A subgroup from Assié et al., 2014.

Genetic alterations	Frequency of patients
Wnt/β-catenin pathway alterations	5/21 (24%)
p53/Rb pathway alterations	4/21 (19%)
Wnt/β-catenin and p53/Rb pathway alterations	8/21 (38%)
Absence of Wnt/β-catenin or p53/Rb pathway alterations	4/21 (19%)

Table S3 – Adrenal Weight by Age

	Control	$PCre^{AS/+}$	$BCre^{AS/+}$	$BPCre^{AS/+}$
1-5 months	3.1±0.7 (n=34)	3.3±0.7 (n=26)	2.8±0.5 (n=7)	3.6±1.5 (n=24)
6-12 months	2.8±1.1 (n=5)	2.9±0.8 (n=13)	9.1±6.3* (n=16)	189.2±229.9** (n=18)

Average Adrenal Weight (mg) Mean±SEM; *P<0.05, **P<0.01; Kruskal–Wallis

Table S4 – Age, Sex and Average Adrenal Weight of the BPCre$^{As/+}$ mice with no tumor (n=26)

	Animal ID	Age (month)	Sex	Adrenal Weight (mg)	Weiss Score	Ki-67 (%)	Metastasis
1	1083996-7	1	F	2.2	-	-	-
2	1083993-1	1	F	1.4	-	-	-
3	1084025-6	1	F	2.9	-	-	-
4	1084084-5	1	F	2.75	-	-	-
5	1084084-8	1	F	2.6	-	-	-
6	1084045b-1	1	F	3	-	-	-
7	1084101-5	1	F	3.1	-	-	-
8	1084022-2	1	F	2.25	-	-	-
9	1084022-5	1	F	1.75	-	-	-
10	377-2	3	F	3.45	-	-	-
11	378-4	3	F	2.5	-	-	-
12	1084045-6	3	F	3.95	-	-	-
13	1803992A-3	3	F	4	-	-	-
14	1084094-1b	3	F	3.75	-	-	-
15	1084071-3	3	F	3.55	-	-	-
16	1084071-4	3	F	3.85	-	-	-
17	1084045A-4	3	M	3.5	-	-	-
18	1084056-7	3	M	3.6	-	-	-
19	389-1	6	M	2.25	-	-	-
20	395-2	5.5	M	7.15	-	-	-
21	393-3b	5	F	4.55	-	-	-
22	393-4	5	F	4.2	-	-	-
23	389-2b	5	M	2.6	-	-	-
24	389-2c	5	M	5.25	-	-	-
25	395-4	5	M	4.35	-	-	-
26	385-5	7	M	1.85	-	-	-

Table S5 – Age Distribution, Sex, Average Adrenal Weight, Weiss Score, Percent Ki-67 and Metastasis of the tumors observed in the BPCre$^{As/+}$ mice (n=16)

	**Animal ID**	**Age (month)**	**Sex**	**Adrenal Weight (mg)**	**Weiss Score**	**Ki-67 (%)**	**Metastasis**
1	4061-7	3.5	F	5.1	2	-	No
2	4094-4	3.8	F	3.55	3	-	No
3	393-6	5.9	F	7.8	1	0.05	No
4	386-2.4	6.5	M	21.3	2	26	No
5	377-7	6.5	F	113.5	4	30	No
6	386-6	6.8	M	23.0	5	16.6	No
7	379-3	7.1	F	9	1	38.53	No
8	379-4e	7.1	F	339	6	12.5	**Yes**
9	387-9e	7.2	M	3.9	1	7.15	No
10	374-1.2	8.0	F	97.9	5	8.91	No
11	383-2	8.4	M	5.3	1	16	No
12	382-1b	9.3	M	258.4	6	27.3	No
13	381-3	9.4	M	80.4	3	10.6	No
14	381-1	9.4	M	423.5	6	14.5	**Yes**
15	396-4	10.4	F	643.5	7	14.7	**Yes**
16	396-7	12.0	F	800	7	45.45	**Yes**

Table S6 – Age Distribution of tumors in BPCre$^{As/+}$ mice

**Age (Month)**	**Weiss<3 ACA**	**Weiss ⩾3 ACC**	**Weiss Score None**	**Total**	**Tumor: ACA or ACC**
<3	0	0	11 (100%)	11	0 (0%)
3-4.5	1 (11%)	1 (11%)	7 (78%)	9	2 (22%)
5-7.5	4 (27%)	3 (20%)	8 (53%)	15	7 (47%)
>7.5	1 (14%)	6 (86%)	0 (0%)	7	7 (100%)

Table S7 – Complete list of probes used in the qPCR experiments.

Probe	Assay ID
Axin2	Mm00443610_m1
Lef1	Mm00550265_m1
Cyp11b2	Mm01204955_g1
Cyp11b1	Mm01204952_m1
Trp53	Mm01731290_g1
Cdkn1a	Mm00432448_m1
Ezh2	Mm00468464_m1
Actb	Mm02619580_g1
Rn18s	Mm03928990_g1

Table S8 – Antibodies

Protein target	Source	Dilution	Application
Beta-catenin	Abcam [12F7] (ab22656)	1:400	IHC
SF-1	Abcam (ab65815)	1:300	IHC
Ki-67	[SP6] (ab16667)	1:300	IHC
Beta-catenin	BD 610153	1:300	IF
